# Effects of Ionizing Radiation on Embryos of the Tardigrade *Milnesium* cf. *tardigradum* at Different Stages of Development

**DOI:** 10.1371/journal.pone.0072098

**Published:** 2013-09-06

**Authors:** Eliana Beltrán-Pardo, K. Ingemar Jönsson, Andrzej Wojcik, Siamak Haghdoost, Mats Harms-Ringdahl, Rosa M. Bermúdez-Cruz, Jaime E. Bernal Villegas

**Affiliations:** 1 Instituto de Genética Humana, Pontificia Universidad Javeriana, Bogotá, Colombia; 2 Department of Molecular Biosciences, The Wenner-Gren Institute, Stockholm University, Stockholm, Sweden; 3 School of Education and Environment, Kristianstad University, Kristianstad, Sweden; 4 Departamento de Genética y Biología Molecular, Centro de Investigación y Estudios Avanzados, CINVESTAV, México D.F, México; UC Irvine, United States of America

## Abstract

Tardigrades represent one of the most desiccation and radiation tolerant animals on Earth, and several studies have documented their tolerance in the adult stage. Studies on tolerance during embryological stages are rare, but differential effects of desiccation and freezing on different developmental stages have been reported, as well as dose-dependent effect of gamma irradiation on tardigrade embryos. Here, we report a study evaluating the tolerance of eggs from the eutardigrade *Milnesium* cf. *tardigradum* to three doses of gamma radiation (50, 200 and 500 Gy) at the early, middle, and late stage of development. We found that embryos of the middle and late developmental stages were tolerant to all doses, while eggs in the early developmental stage were tolerant only to a dose of 50 Gy, and showed a declining survival with higher dose. We also observed a delay in development of irradiated eggs, suggesting that periods of DNA repair might have taken place after irradiation induced damage. The delay was independent of dose for eggs irradiated in the middle and late stage, possibly indicating a fixed developmental schedule for repair after induced damage. These results show that the tolerance to radiation in tardigrade eggs changes in the course of their development. The mechanisms behind this pattern are unknown, but may relate to changes in mitotic activities over the embryogenesis and/or to activation of response mechanisms to damaged DNA in the course of development.

## Introduction

Tardigrades are small aquatic invertebrates known for their high tolerance to extreme conditions, including, e.g., desiccation, freezing and radiation [1], [2]. The physiological and biochemical mechanisms underlying these tolerances are still largely unknown, but many reports have indicated possible mechanisms based on increased activity or accumulation of specific proteins or substances. These include increased levels of enzymes that belong to the ROS system during desiccation [3], induction of heat shock proteins as a response to desiccation [4], [5] and radiation [6], and the presence of late embryogenesis abundant (LEA) proteins in active [7] and in dessicated animals [8].

While tolerance to desiccation and freezing may be explained as evolutionary adaptations promoted by natural selection in habitats that dry out frequently or at a seasonal basis, tolerance to unnaturally high doses of ionizing radiation cannot be viewed as an adaptation. Instead, radiation tolerance is likely to represent a by-product of the adaptive mechanisms evolved to allow survival in dry and cold conditions [9]. Studies on several species of tardigrades have confirmed the extreme tolerance to ionizing radiation of this animal group [10], [11], [12], with an LD_50_ dose of 4–6 kGy (X-ray, gamma, alpha) observed 1–2 days after irradiation. High tolerance also to UV radiation has been reported [13]. This places tardigrades among the most radio-tolerant multi-cellular organisms. For comparison, in *Drosophila melanogaster* the LD_50_/48 h for gamma radiation is around 1300 Gy [14]. Radiation tolerance in tardigrades is not restricted to the desiccated state, but hydrated tardigrades show similar tolerance, which indicates that mechanisms connected to DNA repair may be responsible for the tolerance [10].

Very few studies have been reported on radiation tolerance in tardigrade eggs. However, a recent study evaluated the tolerance of *Ramazzottius varieornatus* eggs to alpha particles (^4^H). Both hydrated and anhydrobiotic eggs were affected by radiation, but hydrated eggs were considerably more sensitive [15]. Exposure of tardigrade eggs (*Milnesium tardigradum*, *Richtersius coronifer*) to space conditions (UV and cosmic radiation, vacuum) led to complete mortality, but when eggs were sheltered from UV radiation, hatchability was not affected [16]. Other studies have shown 100% hatching of *M. tardigradum* eggs exposed to cosmic rays [17]. These studies in space did not evaluate dose responses. Studies evaluating tolerance of tardigrade eggs at different developmental stages are scarce, but an increase in tolerance to desiccation in the course of the embryonic development has been reported in the eutardigrade *M. tardigradum* [18]. A similar result was reported for tolerance to freezing [19]. These studies thus indicate a higher sensitivity of eggs in the initial stage of development. In a recent paper, we showed that tolerance to gamma radiation in eggs of the eutardigrade *R. coronifer* is considerably higher in the late stage of development compared to earlier stages [20]. In the present paper, we report similar results in the eutardigrade *M.* cf. *tardigradum*, but extend the analysis to include both the dose response and the effect of developmental stage.

## Materials and Methods

### Tardigrade culture

For the experiments, cultured specimens of the parthenogenetic eutardigrade *Milnesium* cf. *tardigradum* Doyère, 1840 (Eutardigrada, Apochela, Milnesiidae) were used. The species identity has been preliminarily verified as *M. tardigradum*, but due to lack of morphometric data we follow the recent recommendation of using “cf.” in such cases [21]. Our population originated from moss collected from the roof of a private house at Svinninge, Åkersberga, north of Stockholm, Sweden (N 59°26.88′, E 18°17.43′). The lab population was reared on solid KCM agar plates with a thin layer of distilled milliQ water [18], maintained at room temperature, and fed with rotifers (*Adineta ricciae*, acquired from Department of Chemical Engineering and Biotechnology, Cambridge University). The rotifers were cultured with fish food as energy source. Our study did not involve endangered or protected species, and no permissions were required for collecting animals from the original tardigrade population used in our experiments.

### Experimental setup and monitoring procedures

In tardigrades, egg laying is synchronized with the moulting cycle, and in *M.* cf. *tardigradum* the eggs are laid in the old cuticle (“exuvium”) where they develop into juveniles. In our population, each exuvium contains on average 6 (3–9) eggs. Exuvia with newly laid eggs (in total 450 eggs) were collected and kept in different agar plates, based on day of collection. They were then randomly distributed into 30 small petri dishes with KCM agar, the same as the culturing conditions, with 15 eggs (∼3 or 4 exuvia) per petri dish. To evaluate the sensitivity of tardigrade eggs in the course of development we irradiated eggs at three different developmental stages (early, middle, late). These stages were based on earlier data on egg development in this species [22], and defined as follows: Early stage, from ca. 3 hours to 8 hours post-laying (includes: 2 cells, asynchronous divisions and morula); Middle stage, 43–72 hours (morphogenic movements with visible ventro transversal cleft); Late stage, 120–135 hours (rotatory movements and mouth parts visible). Pictures were taken to verify the stage of the eggs. For each developmental stage, three different irradiation doses were applied; 50, 200 and 500 Gy, using a Cs-137 Gammacell 1000 irradiation source with a dose rate of 6.04 Gy/min (Isomedix, Inc., Kanata, Ontario, Canada). For each of the 9 experimental categories (3 developmental stages×3 doses) and for the controls, 3 replicates were used (giving a total of 9×3+3 = 30 samples). Controls were kept under the same laboratory conditions as the irradiated samples.

After irradiation, eggs were maintained at ambient laboratory temperature (20–22°C) and water was changed every day. The eggs were observed daily under a light microscope for 26 days in order to determine the developmental progress and time of hatching. After the first hatch, rotifers were added to the culture to keep the juvenile tardigrades alive.

### Statistical analyses

Due to small sample sizes we used nonparametric analysis, Mann-Whitney U-test and Kruskal–Wallis one-way analysis of variance by ranks. Reported P-values represent two-tailed tests and P<0.05 was used as criterion for statistical significance.

## Results

### Radiation effects on different developmental stages of *M.* cf. *tardigradum* eggs

**Figure 1:**
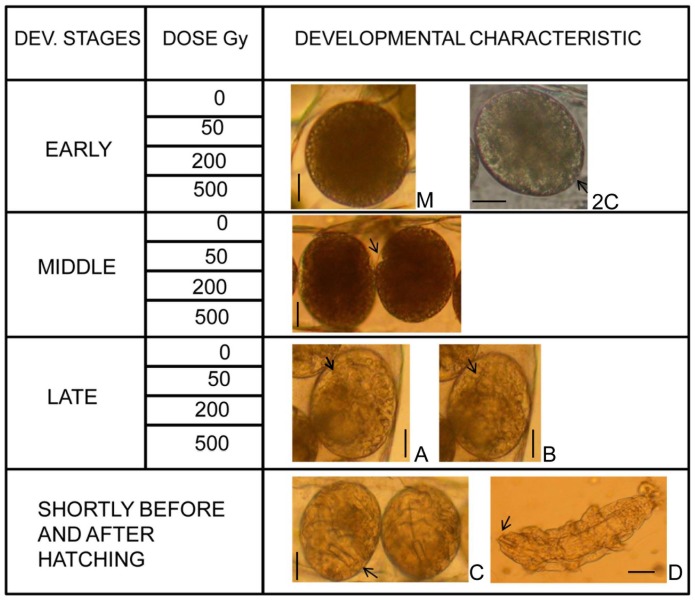
Experimental design with developmental stages, gamma radiation dose levels, and light microscopy photos showing characteristics of embryos in the different developmental stages. The figure shows pictures of non-irradiated embryos representative of the developmental stages used in the experiment. Early stage: Morula (M) and 2-cell stage (2C) of different eggs (arrow indicates the division between the two cells). Middle stage: Formation of the ventro-transversal cleft, indicated by an arrow. Late stage: The rotatory movement caused a slight change in the position of the embryo in the egg. The two pictures are from the same egg with the embryo in different positions, indicated by a structural change from picture A to B in the area pointed at by the arrows. Shortly before and after hatching: Before hatching it is possible to see the buccal tube, indicated by an arrow, which is also visible in the new born larvae. Scale bars in all pictures represent 25 µm.

**Figure 2:**
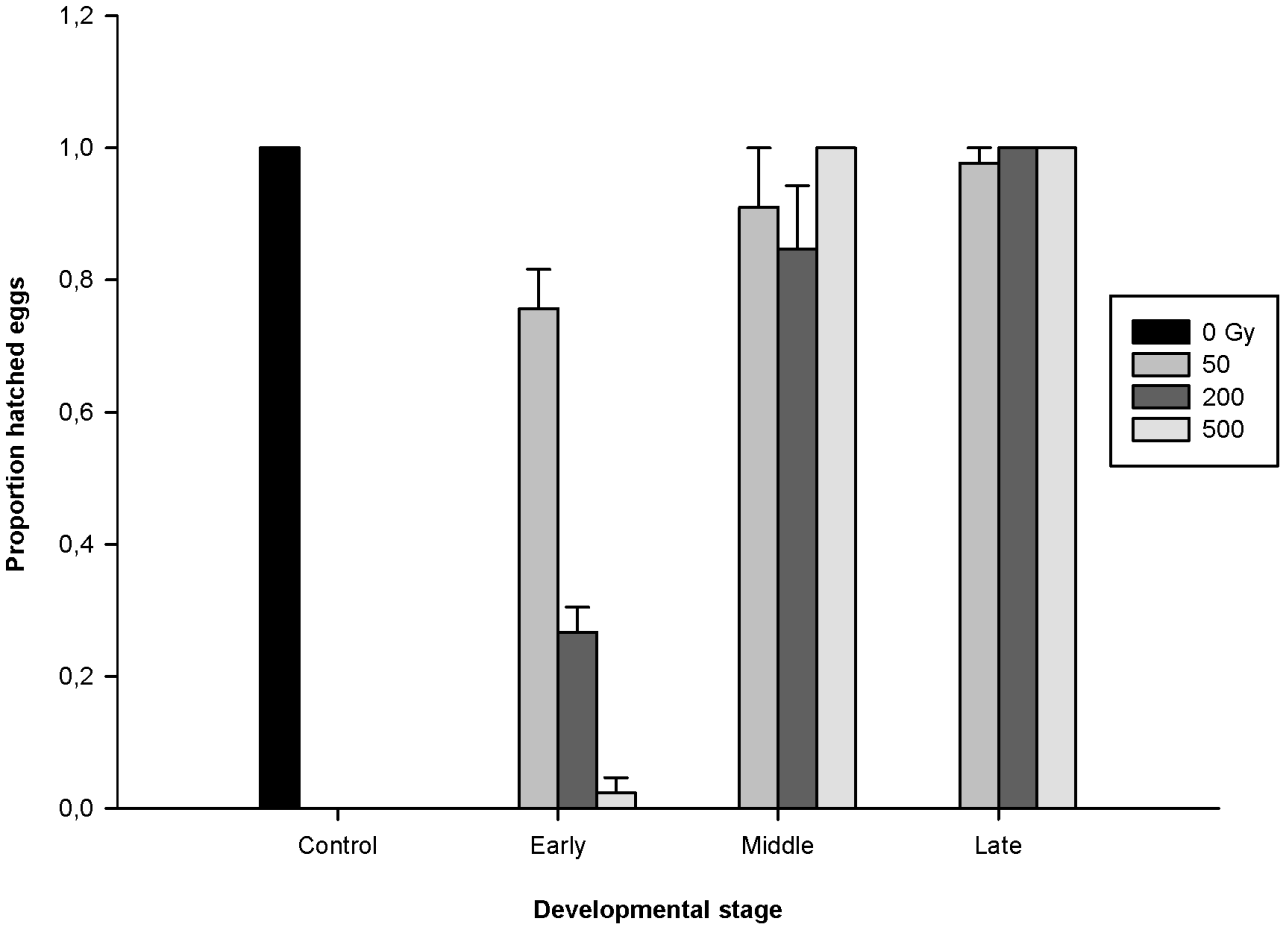
Proportion of hatched eggs of *M.* cf. *tardigradum* eggs exposed to 50, 200 and 500 Gy of gamma radiation, during different developmental stages. Developmental stages are denoted as Early (E), Middle (M) and Late (L). See main text and Fig. 1 for more details on developmental stages. Each bar is based on three replicate samples, each with 15 eggs, and error bars denote standard error of the mean. Estimated mean values (SD): Control, 1.0 (0); E50, 0.76 (0.10); E200, 0.27 (0.067); E500, 0.022 (0.038); M50, 0.91 (0.15); M200, 0.84 (0.17); M500, 1.0 (0); L50, 0.98 (0.038); L200, 1.0 (0); L500, 1.0 (0).

We analyzed the effect of gamma radiation on three different developmental stages of *M.* cf. *tardigradum* embryos (Figures 1, 2). At the lowest dose (50 Gy), we found no significant difference between the three developmental stages (H = 4.28, p = 0.118). In contrast, at both 200 Gy and 500 Gy, the proportion of hatched eggs was significantly or marginally significantly lower in the early developmental stage compared to the middle (200 Gy: U = 0, p = 0.05; 500 Gy: U = 0, p = 0.034) and late (200 Gy: U = 0, p = 0.037; 500 Gy: U = 0, p = 0.034) stage, while the middle and late stage did not differ from each other. These results suggest that eggs are more sensitive to radiation at the early stage of development.

The sensitivity of the early stage was also evident when developmental stages were analyzed separately for dose effects. Only at the early developmental stage there was a significant effect of dose, with lower hatchability at higher doses (H = 10.57, p = 0.014). In this stage 75% of the eggs hatched after a dose of 50 Gy, 26% after 200 Gy, and 2% of the eggs hatched after irradiation with 500 Gy. The dose for 50% mortality (LD50) of the embryos in this stage was estimated (from a linear regression) at about 150 Gy.

### Radiation effects on development time of *M.* cf. *tardigradum* eggs

**Figure 3:**
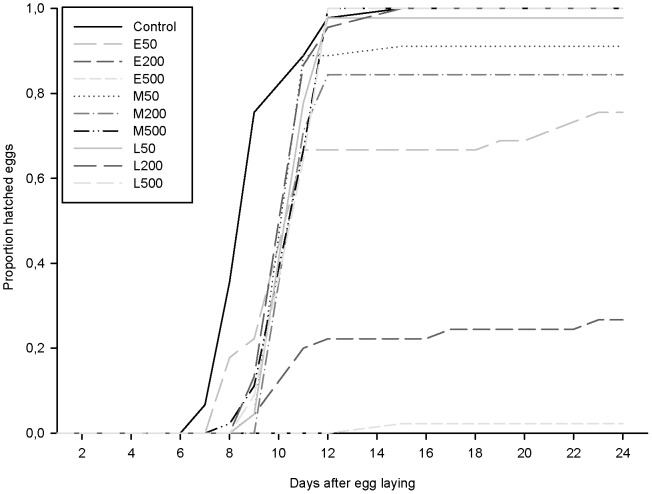
Proportion of hatched eggs as a function of time (days) after egg laying for different experimental groups, defined by developmental stage at irradiation and dose of gamma irradiation. Labels denote developmental stage (E = early, M = middle, L = late) and the dose level (50, 200, 500 Gy). Each line is based on the aggregate total of three replicates, i.e. representing 45 eggs.

We also analyzed development time for the eggs in order to see whether irradiation delayed or advanced development (Figure 3). In the control group the majority of the eggs (76%) hatched after 9 days, while most (64–98%) of the eggs irradiated in the middle and late stage of development hatched after 11 days, with no effect of dose on the pattern of development. Thus irradiation had a clear delaying effect but a high dose did not induce more delay than a low dose. For eggs irradiated in the early stage of development the pattern was different. 22% of the embryos irradiated with 50 Gy hatched on day 9, as in the controls, 45% on day 11 and 9% hatched between day 19 and 22. Of eggs irradiated with 200 Gy 4% hatched on day 9, 16% on day 11, and 7% between day 16 and 24. Only 1 egg was able to hatch after irradiation with 500 Gy, on day 15. These results show that gamma irradiation affects egg development by delaying the hatching time, with no dependence on dose in the middle and late developmental stages but with a tendency of dose-dependence in the early stage.

### Post-hatching observations of juveniles

Observations of animals hatched from the experimental eggs were made every second day until day 36 of the whole experiment (the oldest animals reached an age of 29 days) and although behavior was not estimated quantitatively, these observations provided a qualitative evaluation of the vitality of the animals. The recorded behaviors were general body movements, feeding, and production of eggs. With the exception of juveniles hatched from eggs irradiated with 500 Gy in the early stage of development, the observed behavior was similar for all the stages and all doses, and did not differ compared to the controls. In all groups, animals capturing and eating the rotifer prey were observed, and they also tried to find places to hide within the agar walls. Exuvia with eggs were observed in both controls and irradiated samples. In contrast, the single animal from the early/500 Gy group was clearly affected by the treatment. Although it showed similar body movements in the first 4 days after hatching, it was neither feeding nor molting, and after the 6^th^ day body movements were reduced and it eventually died (8 days after hatching). These observations suggest that, with the exception of the single animal in the early/500Gy group, eggs that managed to hatch after irradiation did not carry any apparent residual effects of the damage.

## Discussion

The results of this study show that extreme tolerance to ionizing radiation in the eutardigrade *M.* cf. *tardigradum* is not a characteristic present from the start of the embryonic development, but is acquired in the course of development. In the early part of development a clear dose response was evident, while in the middle and late stages no such response was observed and the eggs hatched at a rate similar to the control eggs. We have recently found a qualitatively similar pattern in another tardigrade species, *R. coronifer* [20], suggesting that this may be a general characteristic of radiation tolerant tardigrades. These results are also consistent with studies on tolerance to desiccation and freezing in *M. tardigradum*, showing a higher sensitivity to these environmental agents in the early stage of embryo development [18], [19]. For instance, Schill *et al.* [18] showed that eggs in the earliest stage of development had the highest sensitivity to desiccation and that egg sensitivity generally decreased towards the late stages. The results from tolerance experiments with desiccation, freezing, and radiation are therefore consistent with the idea that the tolerance to all these agents is based on the same mechanism [9].

The mechanisms behind the generally high radiation tolerance in tardigrades, as well as the tolerance pattern observed during embryogenesis in our study, are largely unknown. However, a higher sensitivity to radiation of initial stages of embryonic development is expected and is known in radiation biology as the “Law of Bergonié and Tribondeau” [23]. Our study shows that it applies also to the most radiation tolerant animals. The lower tolerance of early developmental stages is generally ascribed to a higher sensitivity of undifferentiated cells and cells undergoing rapid reproduction (mitosis). For example, early stage (preimplantation) mammalian embryos show high sensitivity to radiation [24] and during this phase the embryo is going through the process of DNA replication and cell division until the mid-blastula stage. Irradiation before this stage does not trigger the check point cell cycle responses, even in the presence of DNA damage [24], [25], and DNA damage is therefore not repaired correctly (although the homologous recombination “error free” repair pathway is active in this period). Remaining damage will be amplified by rapid replication and the embryo can progress to the implantation phase, where it is eliminated by apoptosis [24]. A similar mechanism may be hypothesized for the observed sensitivity of tardigrade embryos in the early developmental stage. Few studies on embryological development in tardigrades are available, but Gabriel *et al.* [26] reported that cell division in embryos of *Hypsibius dujardini* declined considerably after 16–17 h post-laying, corresponding to an embryo of approx. 500 cells. The irradiation of the eggs in early developmental stage of our study was made between 3 and 8 hours post-laying, which should be well within the high mitotic activity phase, while the middle and late stages should be well after this phase, taking into account the shorter development time of *H. dujardini* eggs (4–4.5 days). Also the observations made of developing eggs allowed us to confirm stages from 2 cells to morula (Figure 1). Thus the observed sensitivity to radiation in young embryos corresponds to a phase of rapid cell division and is therefore consistent with the “Law of Bergonié and Tribondeau” [23].

Even if tardigrade embryos in early developmental stage are obviously more sensitive to radiation than during later stages, they still show an impressive tolerance. During this stage (2 cells, asynchronous divisions and morula), ∼80% of the eggs survived 50 Gy of gamma radiation, and 20% survived 200 Gy. For comparison, the dose inducing 50% mortality (LD50) initial stage eggs of *C. elegans* has been reported to be 30 Gy [27], while the corresponding value for *M.* cf. *tardigradum* is considerably higher (between 50 and 200 Gy).

We observed a clear delay in embryo development in irradiated samples. Similar delays were reported in tardigrade embryos after desiccation stress [18]. These delays in development may indicate periods of DNA repair, and were most clearly expressed in our study in the middle and late developmental stages. A tendency of dose-response in the developmental delay was observed only in the early developmental stage, which might suggest that the time allowed for repair processes in the middle and late developmental stages was fixed and independent of damage level. In many organisms, including *C. elegans*, *D. melanogaster* and *Xenopus laevis*, early embryogenesis is characterized by rapid progression through the cell cycle [28], [29]. Adherence to the schedule during the initial stage of development (until mid-blastula) seems to be very important for survival of the embryo, and it is programmed by specific signals during development (28). In *Drosophila*, DNA damage during the fast cleavage cycles does not activate the checkpoint response and repair process, which would result in disruption of the developmental program and death, but damage can still disrupt the mitotic chromosome segregation, by inactivation of centrosomes [30]. However, if DNA is damaged during embryo gastrulation (where the G2 phase has been added into cell cycles) it results in a checkpoint-dependent delay of entry into mitosis [30]. Also in the early and fast developing embryos of *C. elegans* DNA damage does not inhibit the cell cycle, and this has been suggested as a mechanism (TLS based) allowing the embryo to survive even when their chromosomes are highly damaged [28]. From the perspective of the radiation tolerance in tardigrades, and also from the fact that tardigrades, nematodes and arthropods belong to the protostome superclade Ecdysozoa it will be of considerable interest to investigate the developmental processes in tardigrades, in particular the checkpoint and DNA repair system.

Our study shows that the tolerance to radiation changes dramatically in the course of the embryological development in the tardigrade *M.* cf. *tardigradum*. The role of cell-cycle checkpoints, DNA repair mechanisms, and apoptosis remains unknown, but the observed delay in development of irradiated embryos may indicate the presence of cell-cycle arrest and connected repair processes, possibly in the middle and late stages of development. There are few molecular studies on embryological stages in tardigrades, but Schokraie *et al.* [31] have reported a study analyzing proteins in the early embryonic stage (corresponding to our early stage), in the active adult stage and in the adult anhydrobiotic stage of *M. tardigradum*. Of 1982 proteins identified in the early embryonic stage, 24% were specific to that stage and of these about 25% were without annotation, but the paper did not refer to or discuss proteins related to DNA repair. Studies in *C. elegans* [27] have indicated that radiation resistance might initially be based on RAD51-mediated homologous recombination as the primary form of repair of double strand breaks in embryos. The involvement of RAD51 also in tardigrades has been indicated in recent work where we observed RAD51 induction in adult *M.* cf. *tardigradum* exposed to gamma radiation [32].

The current study contributes a perspective that has not previously been discussed regarding radiation tolerance in tardigrades, by emphasizing the importance of events during the embryological developmental stage. Obviously, the conditions and mechanisms that allow tardigrades in middle/late embryological stages and in the adult stage to survive high doses of gamma radiations differ from those in the early embryological stage. Whether this difference is mainly due to the vulnerability to radiation of rapidly dividing cells in the early developmental stage, or relates to an onset of an effective DNA repair system at some stage of development remains to be evaluated. The fact that tardigrades are equally tolerant to radiation in the desiccated and hydrated state has led to suggestions that the tolerance relies on a specific and highly efficient DNA repair system [10], but so far the evidence of such system is scarce. However, Neumann *et al.* [33] showed that DNA damage increased over time in desiccated *M. tardigradum*, and also provided some evidence of repair processes after rehydration. Evidence of DNA repair induced by desiccation and radiation has also been reported in some other desiccation and radiation tolerant animals, such as rotifers [34] and insects [35].
